# Polydatin, A Glycoside of Resveratrol, Is Better Than Resveratrol in Alleviating Non-alcoholic Fatty Liver Disease in Mice Fed a High-Fructose Diet

**DOI:** 10.3389/fnut.2022.857879

**Published:** 2022-05-16

**Authors:** Guangshan Zhao, Lian Yang, Wenshen Zhong, Yuze Hu, Yu Tan, Zhe Ren, Qiuyan Ban, Chung S. Yang, Yifei Wang, Zhiping Wang

**Affiliations:** ^1^Guangdong Provincial Engineering Center of Topical Precise Drug Delivery System, School of Pharmacy, Guangdong Pharmaceutical University, Guangzhou, China; ^2^Department of Cell Biology, College of Life Science and Technology, Jinan University, Guangzhou, China; ^3^School of Food Science and Technology, Henan Agricultural University, Zhengzhou, China; ^4^Guangdong Province Key Laboratory of Bioengineering Medicine, Guangzhou, China; ^5^Guangdong Provincial Biotechnology Drug and Engineering Technology Research Center, Guangzhou, China; ^6^Guangzhou Jinan Biomedicine Research and Development Center Co., Ltd., Guangzhou, China; ^7^College of Horticulture, Henan Agricultural University, Zhengzhou, China; ^8^Department of Chemical Biology, Ernest Mario School of Pharmacy, Rutgers, The State University of New Jersey, Piscataway, NJ, United States

**Keywords:** polydatin, resveratrol, non-alcoholic fatty liver disease, 5′-aMP-activated protein kinase, gut microbiota, short-chain fatty acids

## Abstract

Resveratrol (RES) is considered to be an activator of AMP-activated protein kinase (AMPK) with many reported health benefits. Polydatin (POD) is a natural precursor and glycosylated form of RES. The glycoside structure of POD alters the bioactivity. Overnutrition-stimulated reactive oxygen species (ROS) promote the AMPK suppression and metabolic dysregulation. The present work compared the effects of POD and RES in ameliorating energy homeostasis imbalance in mice fed a high-fructose diet and elucidated the underlying mechanisms of action. Our results showed that POD elevated the fecal levels of valeric acid and caproic acid *via* modification of gut microbiota, while RES did not significantly influence the levels of fecal short-chain fatty acids (SCFAs). Both POD and RES markedly decreased the oxidative stress and activated the AMPK signaling pathways in the liver. POD and RES exerted a similar effect in alleviating glucose dysmetabolism, but POD was more effective in ameliorating lipid dysmetabolism than RES. Furthermore, valeric acid and caproic acid alone can activate the AMPK and ameliorate hypercholesterolemia, and enhance the effects of POD on improving lipid metabolism in mice. Overall, for the first time, we demonstrated that POD administration elevated the fecal levels of valeric acid and caproic acid by modifying gut microbiota, thus promoting AMPK activation may be the underlying mechanism that POD is superior to RES in alleviating the lipid dysmetabolism. Our results suggest that POD may be an alternative for RES as an AMPK activator.

## Introduction

**FIGURE 1:**
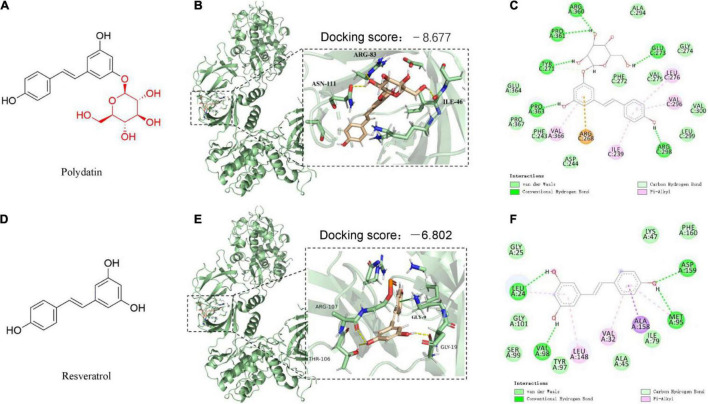
The predicted binding modes of polydatin (POD) and resveratrol (RES) docked into AMP-activated protein kinase (APMK). **(A,D)** Chemical structures of POD and RES, respectively. The binding location of AMPK (black box area) interacting with POD and RES were shown as molecular surface structures in 3D docking model **(B,E)**; H-bonds and hydrophobic interactions between POD or RES and AMPK in a 2D docking model **(C,F)**.

As the glycosylated form of resveratrol (RES, 3,4′,5- trihydroxystilbene) (Figure 1D), polydatin (POD, 3,4′,5-trihydroxystilbene-3-β-D-glucoside) (Figure 1A), also called piceid, is an interesting bioactive compound of Polygonum cuspidatum (2% of dry weight) (1). For a long time, POD was considered to be a lower bioavailability than RES, because the glycoside with a large molecular size and just can be better absorbed when the glycosides are hydrolyzed to their bioactive aglycones RES by β-glucosidases in the small intestine (2–6). *Polygonum cuspidatum* plants, grapes, berries, and peanuts contain RES only approximately 0.2% of dry weight (5), but RES possesses well-known health benefits and is widely applied in medicines, foods, and cosmetic products. RES mainly exists as the glycoside POD in plants (5, 6), hence, to increase the production of RES, POD should be converted into RES by deglycosylation technique (3–6), which is difficult and expensive. In the last decade, accumulated evidence suggests gut microbiota, such as *Lactobacillus* spp., *Bacteroides* spp., and *Bifidobacterium* spp, involves in the absorption, metabolism, and bioavailability of polyphenols glycosides (7–9). For instance, glycosides can be hydrolyzed to bioactive aglycones by β-glucosidases that secreted by bacterial in colon and enable the absorption of polyphenols glycosides (8, 9). *Bifidobacterium* strains show the capacity to enhance the bioavailability of daidzein under dysbiosis conditions (8). In addition, antibiotics treatment caused gut dysbiosis was involved in poor bioconversion of daidzin glycoside and polyphenols (8, 10). These reports indicated that gut microbiota-derived β-glucosidases in the colon play a role in improving the absorption and bioavailability of polyphenols glycosides, especially under the condition that a large amount of ingested but unabsorbed polyphenols glycosides can reach and persist to the colon. Moreover, Wang et al. recently found that POD and RES keep balance through mutual transformation after oral administration and ultimately POD is the main substance in serum (∼70%) (11). Thus, the actual bioactivity of POD and RES *in vivo* needs further comparative investigations.

The prevalence of metabolic disease is a serious public health challenge, as recognized by the World Health Organization in 2000 (12, 13). Fructose consumption increased pronouncedly in recent decades because the use of sucrose and high-fructose corn syrup in processed foods and beverages, and it has markedly contributed to the incidence rate of metabolic disease, such as non-alcoholic fatty liver disease, obesity and type 2 diabetes mellitus (T2DM) (14–16). Mammalian 5′-AMP-activated protein kinase (AMPK) is a regulator of cellular energy homeostasis and a sensor of adenine nucleotides that is activated in states of energy deficiency but suppressed in the overnutrition conditions (17, 18).

Extensive studies have demonstrated that the specific activation of AMPK in liver is beneficial to metabolic syndrome control; thus, AMPK is considered a crucial target for prevention and treatment of overnutrition-associated disease (19–21). Putative AMPK activators, such as the first-line and most prescribed drug metformin, have been identified and developed for the treatment of T2DM (20, 21). SCFAs mainly produced from the catabolism of carbohydrates by gut microbiota (22, 23), and may play an important role in regulating energy homeostasis. Acetate, butyrate, and propionate participate glucolipid neogenesis in liver (24), facilitate fat storage and fatty acid oxidation by browning of fat tissues (25, 26) or restrict energy intake *via* promoting the release of glucagon-like peptide 1 and peptide YY in colon (26, 27). Overnutrition-stimulated ROS promotes the AMPK deactivation by the suppression of phosphorylation of AMPKa at Thr172 (p-AMPKα [Thr172]) is one of the major inducements of metabolic disorders (18, 28, 29).

In addition, numerous studies have shown that nutrient overload could cause the gut microbiota dysbiosis and alter the levels of SCFAs in gut (30–32), and enhance the systemic oxidative stress level (18, 33, 34). All these alterations are consistently associated with the accumulated adipose tissue, elevated body weight, blood glucose level, systemic inflammation, and metabolic complications (30, 35).

Resveratrol is the most widely studied plant-derived natural product that can activate AMPK by multiple mechanisms such as the activation of SIRT1 (36) and the inhibition of mitochondrial function (37) and phosphodiesterases (38). Recent years, several studies suggest that POD possesses higher ability than RES on ameliorating oxidative stress by increasing the levels of total superoxide dismutase (SOD), catalase, glutathione peroxidase, and glutathione and decreasing the level of malondialdehyde (MDA) in mice (11), and has stronger anti-inflammatory effect on reducing the production of proinflammatory cytokine interleukin-17 in human peripheral blood mononuclear cells (39). In addition, POD can prevent fructose-induced liver lipid deposition by activating nuclear factor (erythroid-derived 2)-like 2 antioxidant pathway and scavenging ROS in rats (40), inhibit adipose tissue inflammation and improve the lipid metabolism in high-fat-fed mice (41), and ameliorate glucolipid dysmetabolisms *via* activating AMPK signaling pathway in human hepatoma HepG2 cells (42). Of important is that gut microbiota participates in enhancing the absorption, metabolism, and bioconversion of glycosides by promoting the activity and secretion of β-glucosidases in colon (2, 8, 32). Considering the contribution of accumulated ROS, gut microbiota dysbiosis, and deactivation or suppression of AMPK to energy homeostasis imbalance in the condition of nutrient overload, the above-mentioned reports indicate that POD may possess stronger bioactivity than RES on regulating glucolipid metabolism. Herein, we hypothesized that POD is stronger than RES on ameliorating energy homeostasis imbalance. This study aimed to compare the alleviating effects of POD and RES on glucolipid dysmetabolism and non-alcoholic fatty liver disease and investigate the underlying mechanisms of action in high-fructose diet-fed mice.

## Materials and Methods

### Chemicals and Materials

The primary antibodies AMPKα, p-AMPKα (Thr172), insulin receptor substrate (IRS), p-IRS (Ser307), phosphatidylinositol 3-kinases (PI3K), p-PI3Kp85 (Tyr458)/p55 (Tyr199), protein kinase B (AKT), p-AKT(Ser473), acetyl-CoA carboxylase (ACC), p-ACC (Ser79), thioredoxin-interacting protein (TXNIP) and the second antibodies anti-mouse IgG and anti-rabbit IgG were purchased form the Cell Signaling Technology Inc. (Boston, MA, United States). The primary antibodies peroxisome proliferator-activated receptor-alpha (PPAR-α) and PPAR-β were purchased from the Santa Cruz Biotechnology Co., Ltd. (Dallas, United States) and carnitine palmitoyltransferase-1 alpha (CPT-1α) was obtained from the Abcam (Cambridge, United Kingdom). POD (purity above 95%) and RES (purity above 99%) were obtained from the Aladdin Biochemical Technology Co., Ltd. (Shanghai, China). Fructose, valeric acid sodium and caproic acid sodium were purchased from the Maclin Biochemical Technology Co., Ltd. (Shanghai, China). Recombinant human insulin was bought from Tonghua Dongbao Pharmaceutical Co., Ltd. (Tonghua, China). N-acetylcysteine (NAC) was purchased from the Sigma-Aldrich (St. Louis, MO, United States). Other chemicals and drugs were of the highest grade available.

### Molecular Docking

The isoform and binding site of AMPK (α1β1γ1) was confirmed according to the previously reported AMPK direct activator A-769662 (43–45). The three-dimensional (3D) structure of AMPK was downloaded from the Protein Data Bank (ID: 4ZHX), and the ligand 2D and 3D structures of POD and RES were constructed by Chem3D Ultra (Version 8.0), respectively. The ligand (POD or RES) was docked into the active site of the prepared AMPK crystal structure by Schrodinger (Version 12.5). The binding ability of POD or RES with AMPK was evaluated by docking scores.

### Animal Care Experimental Design for Drug Ddministration

Male C57BL/6J mice, 5 weeks old and weighing 20 ± 2 g, were purchased from the Guangdong Provincial Laboratory Animal Center Co., Ltd. (Guangzhou, China). Mice were housed in a room with controlled temperature (23 ± 2°C, 40 ± 10% relative humidity, and 12-h light–dark cycle), and were allowed free access to water *ad libitum* and food.

**FIGURE 2:**
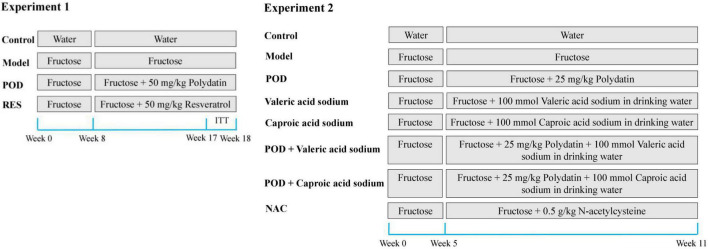
Experimental design for drug administration.

Experiment 1: The animal model of non-alcoholic fatty liver disease was induced by 10% (w/v) fructose for 3 weeks and 30% fructose for 5 weeks in drinking water. The mice were randomly divided into four groups based on body weight (*n* = 5–8): Mice in Control group were given *ad libitum* access to drinking water, mice in Model group were given 30% fructose-containing drinking water, and mice in POD or RES group were given 30% fructose-containing drinking water and 50 mg/kg/day POD or RES by oral gavage, respectively, for another 10 weeks (Figure 2). After that all mice were fasted overnight, peripheral blood was collected from the ophthalmic vein after anesthetized and then the mice were sacrificed by cervical dislocation. Serum was obtained by centrifugation (5,000 round/min, 10 min, 4°C) and stored at −80°C. Livers were collected and stored at −80°C. The fresh fecal samples of each mouse were collected and stored at −80°C during the final week of the animal experiment. POD and RES (5 mg/mL) were suspended sufficiently in the carboxymethylcellulose sodium aqueous solution (0.5%, w/v).

Experiment 2: The animal model of non-alcoholic fatty liver disease was induced by fructose (30%, w/v) in drinking water for 5 weeks. The mice were randomly divided into eight groups based on the body weight (*n* = 5–7): Mice in Control group were given *ad libitum* access to drinking water, mice in Model group were given 30% fructose-containing drinking water, mice in POD group were given 30% fructose-containing drinking water and 25 mg/kg/day POD by oral gavage, mice in valeric acid sodium group were given 30% fructose and 100 mmol/L valeric acid sodium-containing drinking water, mice in caproic acid sodium group were given 30% fructose and 100 mmol/L caproic acid sodium-containing drinking water, mice in POD plus Valeric acid sodium group were given 30% fructose and 100 mmol/L valeric acid sodium-containing drinking water and 25 mg/kg/day POD by oral gavage, mice in POD plus caproic acid sodium group were given 30% fructose and 100 mmol/L caproic acid sodium-containing drinking water and 25 mg/kg/day POD by oral gavage, and the mice in NAC group were given 30% fructose-containing drinking water and 0.5 g/kg/day NAC by oral gavage, for another 6 weeks (Figure 2). After that all mice were fasted overnight and sacrificed by cervical dislocation after anesthetized. Liver and serum were harvested and stored at −80°C. The drinking fluid was exchanged daily.

### Fasting Blood Glucose Measurement and Insulin Tolerance Tests

One touch glucometer (Roche Diagnostics, Mannheim, Germany) was used to measure the fasting blood glucose (FBG) levels on tail vein blood of mice after overnight fasting. For the insulin tolerance tests (ITT), after overnight fasting, insulin (0.3 unit) was administered by the intraperitoneal injection, and the blood glucose was measured at 0.5, 1.0, 1.5, and 2.0 h after the injection with the glucometer.

### Serum Parameters Assay

Serum aspartate aminotransferase (AST), alanine aminotransferase (ALT), creatinine (Cr), and blood urea nitrogen (BUN) levels were measured with an automatic biochemical analyzer (HITACHI 7020, Japan). Kits for triglyceride (TG), total cholesterol (TC), free fatty acid (FFA), LDL-cholesterol (LDL-C), MDA, and SOD obtained from Nanjing Jiancheng Bioengineering Institute (Nanjing, China). The levels of serum insulin and HbA1c were measured using the commercial ELISA kits from Jiangsu Meimian industrial Co., Ltd. (Yancheng, China) and Jiangsu Boshen Biotechnology Co., Ltd. (Nanjing, China). Homeostasis model assessment-insulin resistance (HOMA-IR) index was calculated by the following formula:


HOMA-IR=Glucose(mmol/L)×Insulin(mU/L)÷22.5

### Quantitation of Total Cholesterol, Triglyceride, and Free Fatty Acid in Liver

Livers (0.1 g) were homogenized in ice-cold tissue extraction buffer (1 ml) and then the lysates were clarified by centrifugation (12,000 *g*, 4°C, 15 min), after that the supernatant was collected for analysis. The levels of TC, TG, and FFA were measured according the corresponding protocols in the commercial kits provided by manufacturers.

### Histology Assay

Harvested liver specimens were fixed in paraformaldehyde solution at room temperature, dehydrated, embedded in paraffin, after that a fully automated rotary microtome (LEICA RM2255, Shanghai, China) was used to cut the paraffin into 5 μm thickness serially. Periodic acid-schiff (PAS), hematoxylin-eosin (HE), MASSON, and oil red O stains were performed according to the standard protocols of the corresponding commercial kits, respectively, and then the slices were pictured with electron microscope (Nikon eclipse ti, Japan). All the assays were performed in a blinded manner.

### Quantitative Real-Time Polymerase Chain Reaction

Total RNA was extracted from liver tissues of mouse using TRIzol reagent, obtained from Takara Biotechnology, according to the manufacturer’s protocol. The cDNA was prepared using 50 ng of total RNA by the reverse transcription according to the manufacturer’s instructions. SYBR Green qPCR SuperMix was performed on a CFX System (Bio-Rad, Hercules, CA, United States) according to the manufacturer’s instructions. Real-time PCR of cDNA was performed using standard PCR cycling condition. Relative expression level of target gene was normalized against control group β-actin and presented as a ratio to the expression level in other groups with the formula 2^–(ΔΔ*Ct)*^. The primer sequence of each tested gene is shown in Supplementary Table 1.

### Western Blot

Livers (0.1 g) were homogenized in 0.1 mg/ml phenylmethylsulfonyl fluoride-containing ice-cold RIPA buffer (1 ml) and then the lysates were clarified by centrifugation (12,000 *g*, 4°C, 15 min), after that the supernatant was collected and stored at −80°C. The samples were denatured with loading buffer (99°C, 10 min), then the equal protein were separated by SDS-PAGE gel electrophoresis and transferred onto a polyvinylidene fluoride (PVDF) membrane. The membrane was probed with primary antibody according to the dilution ratio provided by manufacturers overnight at 4°C, and then incubated with secondary antibody according to the dilution ratio provided by manufacturers at room temperature for 60 min. The immunoreactivity was detected using the ChemiDoc XRS + detection system (ECL, Bio-Rad, United States). The densitometric analysis was performed with Quantity One^®^ Image Analyzer software program (Bio-Rad). Glyceraldehyde-3-phosphate dehydrogenase (GAPDH) was used for normalization.

### Gut Microbiota Profiling

Total genome DNA of bacterial was extracted from frozen feces with QIAamp DNA stool Mini Kit (Qiagen, Hilden, Germany) according to the manufacturer’s guideline. The specific primer with the barcode (16S V3 + V4) was used to amplify the 16S rDNA gene. TruSeq^®^ DNA PCR-Free Sample Preparation Kit (Suzhou RENOLD Biological Technology Co., Ltd., Suzhou, China) was used to construct the DNA sequencing libraries. Fast Hifidelity Polymerase and Phusion^®^ High-Fidelity PCR Master Mix with GC Buffer (New England Biolabs Co., Ltd., Beijing, China) were used for The PCR amplification under the standard thermal cycling and extension conditions. Paired-end sequencing of the PCR products was performed on the NovaSeq6000 at Suzhou Bionovogene Co., Ltd. (Suzhou, China).

### Short-Chain Fatty Acids Measurement

Gas chromatography-mass spectrometer method (Thermo TRACE 1310-ISQ LT instrument, Agilent HP-INNOWAX column with 30 m × 0.25 mm ID × 0.25 μm particle size) (Suzhou Bionovogene Co., Ltd., China) was used to measure the fecal levels of acetic acid, propionic acid, butyrate, isobutyric acid, caproic acid, valerate and isovaleric acid. 50 μL phosphoric acid (15%), 100 μL internal standard (isohexic acid) solution (125 μg/mL) and 400 μL ether were homogenated with 50 mg fresh feces for 1 min, then the mix were centrifuged at 12000 rpm and 4°C for 10 min. After that the supernatant was collected for measurement.

### Statistical Analysis

Data were expressed as mean ± SEM, and compared using the Student’s *t*-test or two-way ANOVA *post hoc* Bonferroni test, as appropriate (GraphPad Prism 5 Software, Inc., La Jolla, CA, United States). The correlation coefficient between the fecal levels of SCFAs and the relative abundance of gut microbiota at the genus level were performed with Pearson correlation analysis (SPSS software, version 20, IBM, Armonk, NY, United States). The significance levels were established at a *p*-value of <0.05.

## Results

### Binding Interactions of Polydatin and Resveratrol With Activated Protein Kinase

To better understand the interactions of POD and RES with AMPK, molecular docking study was performed to analyze the binding abilities of POD and RES with AMPK. The molecular docking results between the ligands (POD and RES) and the receptor (AMPK) suggest POD displayed a higher predicted binding score than RES (−8.677 *vs*. −6.802) (Figures 1B,E). This indicates that POD possesses a stronger interaction with AMPK than RES. Conventional hydrogen bond and van der Waals are the mainly binding bonds between POD and RES with AMPK. POD exerts higher binding ability with AMPK than RES may involve there are more hydroxyl groups in POD (Figure 1) because hydroxyl groups formed H-bonds to AMPK (Figure 1C) are important for stable binding. The more residues of AMPK units or binding pocket involved in the H-bond interaction or the binding of POD and AMPK by van der Waals also contributed to its binding (Figure 1C).

### Polydatin Is More Active Than Resveratrol in Alleviating Lipid Dysmetabolism Than Resveratrol in Mice Fed a High-Fructose Diet

**FIGURE 3:**
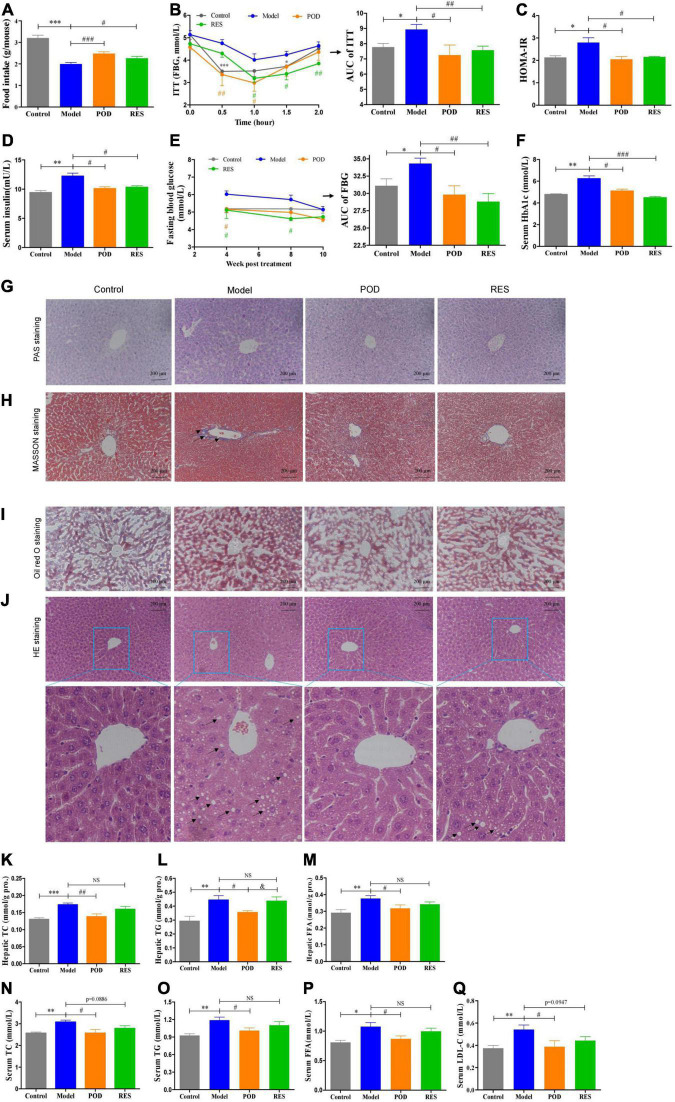
Effects of POD and RES on glucolipid metabolism and fatty liver. **(A)** Food intake. **(B)** Insulin tolerance test (ITT) and the area under the curve (AUC). **(C)** Homeostasis model assessment -insulin resistance (HOMR-IR, HOMA-IR = Glucose [mmol/L] × Insulin [mU/L] ÷ 22.5). **(D)** Serum insulin level. **(E)** Fasting blood glucose level (FBG) and AUC of FBG. **(F)** Serum HbA1c level. **(G–J)** Hepatic periodic acid-schiff (PAS) (200×), MASSON (200×), Oil red O (200×), and HE stainings (200×), respectively. **(K–M)** Total cholesterol (TC), triglycerides (TG), and free fatty acid (FFA) in liver, respectively. **(N–Q)** Serum levels of TC, TG, FFA, and low-density lipoprotein-cholesterol (LDL-C), respectively. Data are presented as mean ± SEM. * *p* < 0.05, ^**^ *p* < 0.01, ^***^ *p* < 0.001. ^#^ *p* < 0.05, ^##^ *p* < 0.01, ^###^ *p* < 0.001, compared to model group. ^&^ *p* < 0.05, compared to POD group. NS, *p* > 0.05.

Body weight (Supplementary Figure 1A) and serum parameters, such as ALT, AST, Cr, and BUN (Supplementary Table 2), showed that the doses of POD and RES employed did not possess toxicity in mice. Since the intake of extra calories from fructose in drinking fluid (Supplementary Figure 1B), the food intake was lowered (Figure 3A). Both the POD and RES administrations did not significantly influence the fluid intake (Supplementary Figure 1A), but, interestingly, elevated the food intake of the mice (Figure 3A). High-fructose diet enhanced the insulin tolerance (Figures 3B,C), elevated the levels of serum insulin, FBG, and serum HbA1c (Figures 3D–F) and caused the hepatic glycogen accumulation and fibrogenesis (Figures 3G,H). POD and RES alleviated these lesions with a similar effect (Figures 3D–H). In addition, serious lipid accumulation (Figure 3I), fatty infiltration and amyloidosis (Figure 3J) of liver were also observed in the model mice. POD showed a better effect on preventing these lesions than RES (Figures 3I,J). This was also validated by the higher capacity of POD in reducing the levels of TC and TG in liver (Figures 3K,L) and serum (Figures 3M,N). These results suggest that POD and RES can alleviate the glucolipid dysmetabolism, and POD is superior to RES on ameliorating non-alcoholic fatty liver disease than RES in mice fed a high-fructose diet.

**FIGURE 4:**
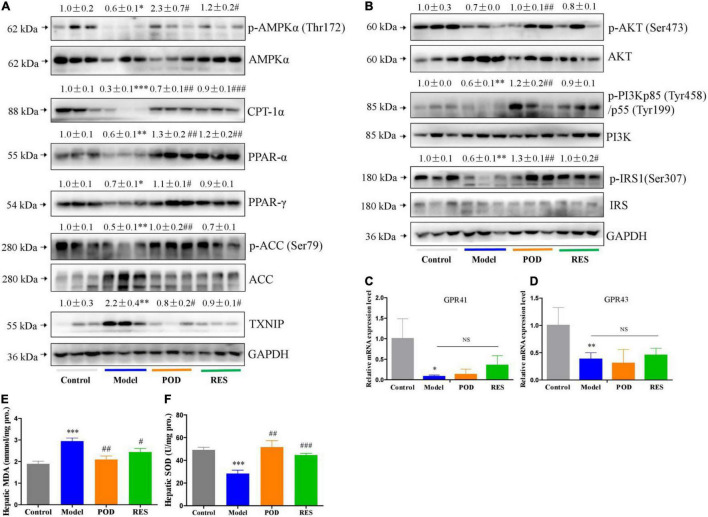
Effects of POD and RES on AMPK signaling pathway and biomarkers of oxidative stress in liver. **(A)** AMPK signaling pathway proteins. **(B)** Insulin signaling pathway proteins. **(C,D)** The mRNA levels of GPR41 and GPR43. **(E)** MDA level. **(F)** SOD enzyme activity level. Data are presented as mean ± SEM. * *p* < 0.05, ^**^ *p* < 0.01, ^***^ *p* < 0.001, compared to control group. ^#^ *p* < 0.05, ^##^ *p* < 0.01, ^###^ *p* < 0.001, compared to model group. NS, *p* > 0.05.

At the molecular level, fructose consumption suppressed the AMPK signaling pathway, such as downregulation of p-AMPK (Thr172), CPT-1α, PPAR-α, PPAR-γ, p-ACC (Ser79), p-AKT (Ser473), -PI3Kp85 (Tyr485), and p-IRS1 (Ser307) and upregulation of TXNIP, in liver (Figures 4A,B). POD significantly prevented these alterations (Figures 4A,B), and RES displayed a preventing effect on the downregulation of p-AMPKα (Thr172) (Figure 4A), but without affecting the levels of the downstream proteins PPAR-γ, p-ACC (Ser79), p-AKT (Ser473), and p-PI3Kp85 (Tyr485) (Figures 4A,B). In addition, long-term fructose consumption caused elevation of oxidative stress level in liver indicated by the pronouncedly increase of TXNIP (Figure 4A) and MDA (Figure 4E) and decrease of SOD (Figure 4F), both POD and RES administrations effectively prevented these alterations (Figures 4E,F). However, neither POD nor RES affected the mRNA levels of GPR41 and GPR43 in liver (Figures 4C,D).

### Polydatin Increases the Fecal Levels of Caproic Acid and Valeric Acid by Modification of the Gut Microbiota

**FIGURE 5:**
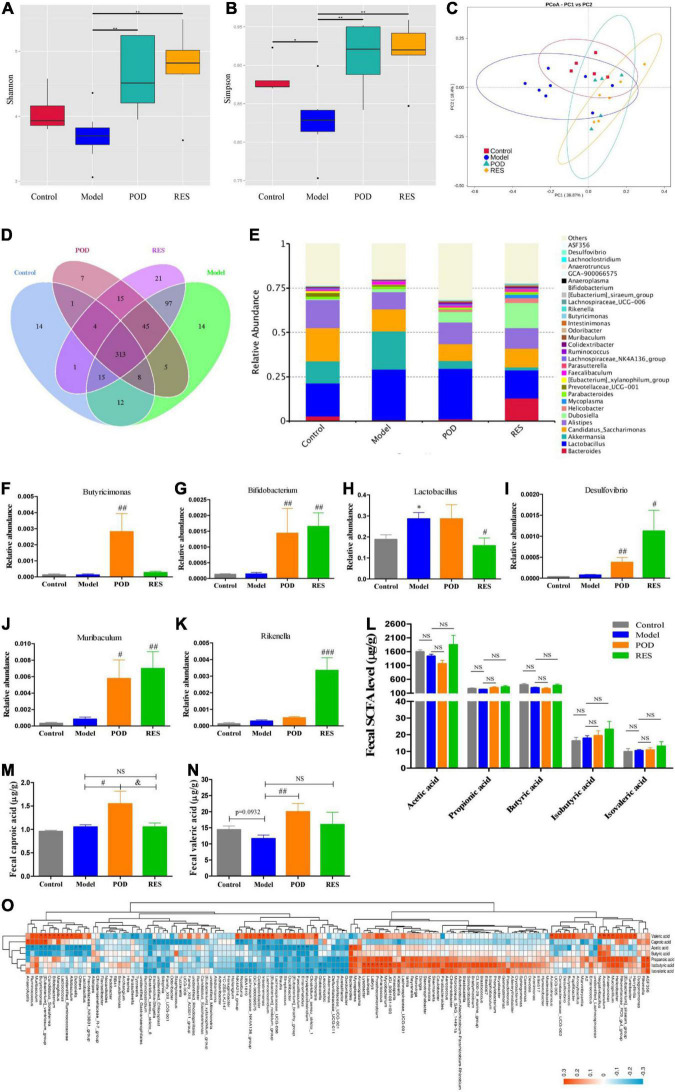
Microbiome and SCFAs of the fecal samples. Alpha-Diversity was presented by a box plot of the Shannon **(A)** and Simpson **(B)**. **(C)** Principal coordinate analysis (PCoA) plot analysis. **(D)** Petal analysis of OTU. **(E)** Relative abundance of gut microbiota at the genus level (top 30). **(F–K)** Relative abundance of the altered microbiota. **(L–N)** Levels of SCFAs. **(O)** Correlation analysis of SCFAs level and gut microbiota abundance at the genus level. Data are presented as mean ± SEM. Orange, positive correlation. Blue, negative correlation. * *p* < 0.05, ^**^ *p* < 0.01, compared to control group. ^#^ *p* < 0.05, ^##^ *p* < 0.01, ^###^ *p* < 0.001, compared to model group. ^&^ *p* < 0.05, compared to POD group. NS, *p* > 0.05.

The α-diversity analysis of the gut microbiomes showed frcutose consumption lowered the diversity of microbiota (Figures 5A,B). POD and RES administrations markedly increased the microbiota diversity evidenced by the elevated Shannon and Simpson indexes (Figures 5A,B). The β-diversity analysis evaluated the overall differences in groups, results showed a distinct clustering of gut microbial community structure in control and model groups (Figure 5C), POD and RES administrations altered the structure in a similar trend (Figure 5C). The Venn diagrams were used to show the over lapping operational taxonomic units (OTUs), which displayed the similarity and consistency of samples. There are 313 OTUs shared in all groups (Figure 5D). Three hundred and 68 OTUs were identified in control group, and fructose consumption increased the OTUs numbers to 509 (Figure 5D). Interestingly, POD administration lowered the OTUs numbers to 398 and RES increased the OTUs numbers to 511 (Figure 5D). These results indicated long-term intake of fructose markedly affected the community structure and relative abundance of gut microbiomes (Figures 5A–E), and POD has a higher regulating effect on gut microbiomes than RES (Figure 5D). The function of the significantly altered gut microbiota (Figures 5F–K) post POD or RES treatment will be discussed in the discussion section.

Our results showed the levels of fecal SCFAs of mice in the model group without a significant alteration compared to control (Figures 5L–N), POD treatment markedly elevated the levels of valeric acid and caproic acid (Figures 5M,N), but RES did not pronouncedly affect the levels of SCFAs (Figures 5L–N). Correlation analyses between SCFAs levels and relative abundance of gut microbiota indicated the valeric acid level was significantly negative correlated with the abundance of *Parasutterella* (Figure 5O) and positive correlated with the abundance of *Acetitomaculum*, *Anaerotruncus*, *Candidatus_Soleaferrea*, *Colidextribacter*, *Harryflintia*, *Mucispirillum*, *Negativibacillus*, *Butyricimonas*, *[Eubacterium]_ventriosum_group*, *Candidatus_Soleaferrea*, *Bifidobacterium*, *Monoglobus*, *Lactococcus*, *Allobaculum*, *Intestinimonas*, *Lachnoclostridium*, *[Eubacterium]_nodatum_group*, *Streptococcus*, *Romboutsia*, *Anaerovorax*, and *unidentified_Ruminococcace* at the genus level (Figure 5O). However, the caproic acid level was significantly negative correlated with the abundance of *Escherichia-Shigella*, *NK4A214_group*, *Roseburia* and *Colidextribacter*, and positive correlated with the abundance of *Anaerofustis* and *[Eubacterium]_ventriosum_group* (Figure 5O).

### Caproic Acid and Valeric Acid Activate Activated Protein Kinase and Enhance the Effects of Polydatin on Alleviating Lipid Dysmetabolism in Mice Fed a High-Fructose Diet

**FIGURE 6:**
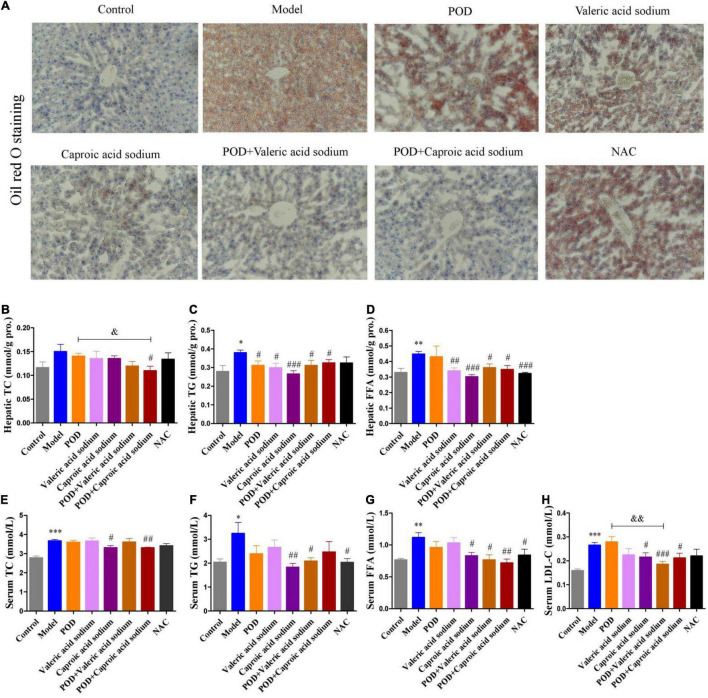
Effects of valeric acid and caproic acid on lipid metabolism in mice fed a high-fructose diet. **(A)** Oil red O staining (200 ×). **(B–D)** Levels of TC, TG, and FFA in liver. **(E–H)** Levels of TC, TG, FFA, and LDL-C in serum. Data are presented as mean ± SEM. * *p* < 0.05, ^**^ *p* < 0.01, ^***^ *p* < 0.001, compared to control group. ^#^ *p* < 0.05, ^##^ *p* < 0.01, ^###^ *p* < 0.001, compared to model group. ^&^ *p* < 0.05, ^&&^ *p* < 0.01, compared to POD group. NS, *p* > 0.05.

**FIGURE 7:**
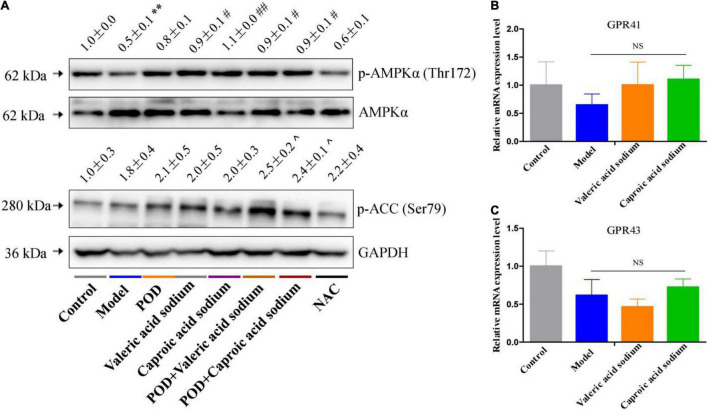
Effects of valeric acid and caproic acid on p-AMPK α (Thr172) and p-ACC (Ser79) proteins and GPR41 and GPR43 mRNA levels in liver. **(A)** The protein levels of p-AMPK α (Thr172) and p-ACC (Ser79). **(B,C)** The mRNA levels of GPR41 and GPR43. Data are presented as mean ± SEM. ** *p* < 0.01, compared to control group. # *p* < 0.05, ## *p* < 0.01, compared to model group. ^ *p* < 0.05, compared to control group. NS, *p* > 0.05.

The markedly elevated levels of fecal valeric acid and caproic acid *via* modifying gut microbiomes by POD administration may be the main reason that POD exerted a higher property on ameliorating lipid dysmetabolism than RES. Thereby, we next investigated the regulating effects of valeric acid and caproic acid on AMPK and lipid metabolism in mice fed a high-fructose diet. Results showed that both caproic acid sodium and valeric acid sodium reduced the lipid accumulation, and the levels of TG and FFA in liver (Figures 6A,C,D). In addition, caproic acid sodium decreased the levels of TC, TG, FFA, and LDL-C in serum (Figures 6E–H). Importantly, we found when the low dose of POD (25 mg/kg) without a pronouncedly improvement on non-alcoholic fatty liver disease, caproic acid sodium and/or valeric acid sodium can enhance the effects of POD on preventing lipid dysmetabolism (Figures 6A,B,F–H). Consistently with these improving effects of SCFAs (caproic acid sodium and valeric acid sodium) or SCFA plus POD on lipid metabolism, AMPK was activated post the treatment indicated by the significantly upregulation of p-AMPK α (Thr172) and p-ACC (Ser79) (Figure 7). Neither caproic acid sodium nor valeric acid sodium affected the mRNA levels of GPR41 and GPR43 in liver.

## Discussion

In this work, we firstly demonstrated that POD shows higher ameliorating effect on non-alcoholic fatty liver disease than RES in mice fed a high-fructose diet. The mechanism of action may involve POD administration promoted the elevation of valeric acid and caproic acid in feces by modifying gut microbiota thus activating the AMPK signaling pathway. Since nutrition overload and overnutrition-stimulated ROS promoted the AMPK deactivation or suppression which is the major pathological mechanism of metabolic disorders (17, 18, 28, 29) (Figures 4A, 7A), considering POD has been proved with the property of activating AMPK *in vitro* (42) and scavenging ROS *in vivo* (40, 46, 47), and exerts stronger anti-oxidant capacity *in vivo* (11) and anti-inflammatory effect *in vitro* than RES (39). Our results suggest that POD displayed a higher effects than RES on improving the overnutrition-related diseases by activating the AMPK signaling pathway *via* modification of the gut microbiota *in vivo*.

Gut microbiota and its metabolites SCFAs and oxidative stress state play an important role in maintaining energy homeostasis through regulating AMPK. *Butyricimonas* is a SCFA-producing bacteria (48–52) and participates in driving the reduction of body mass index (BMI) in response to insulin (50, 52). POD treatment pronouncedly increased the abundance of *Butyricimonas* (Figure 5F) though the fructose intake without significant decreasing the abundance of *Butyricimonas* at the genus level. *Bifidobacterium* is considered a probiotics that participates SCFAs production (8, 9, 53). Both POD and RES markedly increased the abundance of *Bifidobacterium* at the genus level (Figure 5G). *Lactobacillus* involves in the absorption and metabolism of polyphenols glycosides by enhancing the activity and secretion of β-glucosidase (7–9). POD did not compromise the abundance of Lactobacillus, but RES exerted a suppression effect on *Lactobacillus* when fructose consumption increased the abundance of *Lactobacillus* at the genus level (Figure 5H). *Desulfovibrio*, *Muribaculum*, and *Rikenella* are probiotics that beneficial for the energy homeostasis (8, 54, 55). Both POD and RES significantly increased the abundance of the genera *Desulfovibrio* and *Muribaculum* (Figures 5I,J), and RES increased the genera *Rikenella* (Figure 5K). In addition, correlation analyses between SCFAs levels and gut microbiota abundance indicated that the valeric acid level was significantly positive correlated with the abundance of *Butyricimonas*, *[Eubacterium]_ventriosum_group* and *Bifidobacterium* (Figure 5O). And the caproic acid level was significantly positive correlated with the abundance of [Eubacterium]_ventriosum_group (Figure 5O). These results suggest that *Butyricimonas*, *[Eubacterium]_ventriosum_group* and *Bifidobacterium* may involve in the elevation of fecal levels of valeric acid and caproic acid (Figures 5M,N). Of interest is that POD displayed a higher regulating effect on the gut microbiomes and its metabolites SCFAs than RES, which is indicated by the reversed OTUs (Figure 5D) and pronouncedly elevated the valeric acid and caproic acid levels in feces (Figures 5M,N) by POD administration. This is in line with the reports that since the ingested glycosides are poorly absorbed by the small intestine, a significant fraction of POD can persist to the colon, where they encounter the gut microbiota and play a better modifying effect on the structure and composition of microbiota and ultimately affecting its metabolites (2, 8, 32).

Emerging evidence indicates that intestinal microbial metabolites influence the host and contribute to the development of metabolic syndrome and T2DM (24, 56). SCFA formed from the result of a complex interplay between the gut microbiota and dietary fiber. As the signaling molecules between the gut microbiota and the host, SCFAs play a regulatory role on human metabolism in local, intermediary, and peripheral metabolism (56). As the endogenous receptors for SCFAs, G protein-coupled receptor free fatty acid receptor 2 (FFAR2, GPR43), and FFAR3 (GPR41) have already been identified. Acetate, propionate, and butyrate are the most abundant SCFAs produced by microbiota and presented in the gut lumen at high levels (57–59). Meanwhile, the shorter acetate preferentially activates GPR43, the longer butyrate preferentially activates GPR41, and propionate displays similar agonism on GPR43 and GPR41 (60). However, several reports have clarified and discussed that SCFAs can activate the AMPK and maintain the energy homeostasis (61–63) in a GPR43 or GPR41 dependent (64–66) or independent (63, 67) mechanism, and the independent mechanism is consistent with the finding that butyrate and propionate still ameliorated insulin resistance and body weight gain in GPR41-deficient mice (67). In this study, we found the markedly elevated fecal levels of valeric acid and caproic acid by modification of gut microbiota by POD administration can activate the AMPK (Figures 4A, 7A) and promote the effects of POD on ameliorating lipid dysmetabolism (Figures 6A,B,F–H) without affecting the mRNA levels of GPR43 and GPR41 in liver (Figures 4C,D, 7B,C). Our results firstly demonstrated the activation of valeric acid and caproic acid on AMPK *in vivo*, and reinforced the concept that SCFAs activate AMPK is likely the common mechanism (61, 63, 67) for alleviating the energy homeostasis imbalance in a GPR43 or GPR41 independent manner.

The NAC, recognized as a ROS scavenger, is widely employed as a tool for explaining the consequences of oxidative stress and as a clinical drug for antioxidant therapy (68, 69). Scavenging of ROS is beneficial for alleviating metabolic disorders in high glucose conditions (18, 70). Indeed, in the present work, we found NAC administration significantly reduced the levels of FFA in liver (Figure 6D) and TG and FFA in serum (Figures 6F,G) in mice fed a high-fructose diet. However, NAC treatment without preventing the downregulation of p-AMPK α (Thr172) (Figure 6A), and the elevation of lipid accumulation, TC and TG in liver (Figures 6A–C), and TC and LDL-C in serum (Figures 6E,H). This indicates that reducing the level of oxidative stress alone by NAC cannot reverse the lipid dysmetabolism induced by high-fructose diet. POD administration alleviated oxidative stress by reducing ROS-driven TXNIP over-expression (Figure 5A) (40) and lipid peroxide MDA (Figure 4E), and enhancing the antioxidant enzyme SOD in liver (Figure 4F); and activated hepatic AMPK signaling pathway (Figures 4A, 7A) by elevating the fecal levels of valeric acid and caproic acid *via* modifying gut microbiota (Figures 5M,N). As a consequence of the improved oxidative stress state and activated AMPK signaling pathway, POD ameliorated the lipid dysmetabolism more effectively (Figure 3). Furthermore, the crucial role of valeric acid and caproic acid in activation of AMPK and improvement of lipid metabolism were confirmed by the upregulation of p-AMPK upalpha (Thr172) (Figure 7A) and the reduction of lipid accumulation, TC, TG, FFA, or LDL-C in liver and serum (Figure 6) by valeric acid sodium and/or caproic acid sodium treatment and valeric acid sodium or caproic acid sodium plus POD administration in mice fed a high-fructose diet.

## Conclusion

**FIGURE 8:**
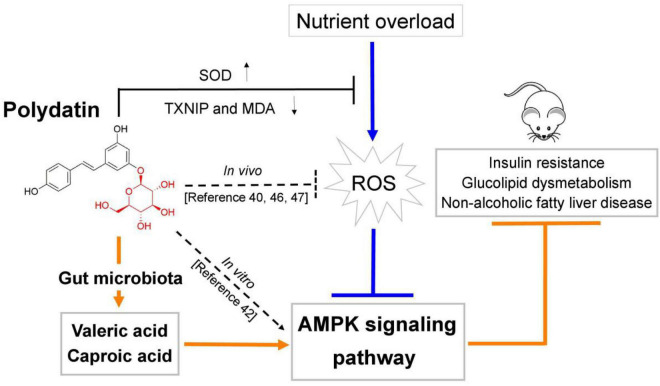
Schematic diagram showing the underlying mechanisms of polydatin administration on ameliorating energy homeostasis imbalance.

In summary, this study investigated the ameliorating effects of POD and RES on insulin resistance, glucolipid dysmetabolism and non-alcoholic fatty liver disease by reducing the oxidative stress and preventing AMPK suppression induced by high-frcutose diet in mice (Figure 8). For the first time, we found POD possesses a higher improvement effect on non-alcoholic fatty liver disease than RES (Figures 3I–Q) in mice, the mechanism of action may involve the pronouncedly elevated fecal levels of valeric acid and caproic acid *via* modification of gut microbiota by POD administration can activate AMPK signaling pathway and enhance the effects of POD on alleviating lipid dysmetabolism (Figures 4A, 7A, 6A,B,F–H). RES is considered to be an AMPK activator (71–73) with well-known health benefits and widely applied in medicines, foods, and cosmetic products. As a natural precursor of RES, POD is superior to RES in anti-oxidant (11), anti-inflammatory (39), modification of gut microbiota (Figures 5D,M,N), and improvement of lipid metabolism (Figures 3I–Q). Thus, POD may be an alternative of RES as AMPK activator and for industrial and medical applications.

## Data Availability Statement

The datasets presented in this study can be found in online repositories. The names of the repository/repositories and accession number(s) can be found below: NCBI; PRJNA799661.

## Ethics Statement

The animal study was reviewed and approved by Institutional Animal Care and Use Committee at Jinan University (IACUC Issue No: 20200329-22).

## Author Contributions

GZ: conceptualization. GZ, LY, QB, and YH: methodology. GZ, LY, WZ, and YT: investigation. GZ, ZW, and YW: validation, project administration, and funding acquisition. GZ, QB, and LY: writing—original draft preparation. GZ, CY, and QB: writing—review and editing. GZ, ZW, YW, and ZR: supervision. All authors contributed to the article and approved the submitted version.

## Conflict of Interest

GZ, ZR, and YW were employed by the company Guangzhou Jinan Biomedicine Research and Development Center Co., Ltd. The remaining authors declare that the research was conducted in the absence of any commercial or financial relationships that could be construed as a potential conflict of interest.

## Publisher’s Note

All claims expressed in this article are solely those of the authors and do not necessarily represent those of their affiliated organizations, or those of the publisher, the editors and the reviewers. Any product that may be evaluated in this article, or claim that may be made by its manufacturer, is not guaranteed or endorsed by the publisher.
