# Supplementary material for: Polydatin, A Glycoside of Resveratrol, Is Better Than Resveratrol in Alleviating Non-alcoholic Fatty Liver Disease in Mice Fed a High-Fructose Diet
Source: Front Nutr. 2022 May 16;9:857879. doi: 10.3389/fnut.2022.857879 (PMC9149290; doi:10.3389/fnut.2022.857879)
Supplement: Supplementary file 1 [file Data_Sheet_1.docx]

Supplementary Material

# Supplementary Table 1. Gene-specific primers.

| **Genes** | **Direction** | **Sequences** |
| --- | --- | --- |
| GPR41 | Forward | 5ˊ-CCACACTGCTCATCTTCTTCGTCTG-3ˊ |
|  | Reverse | 5ˊ-ACGGACTCTCACGGCTGACATAG-3ˊ |
| GPR43 | Forward | 5ˊ-CTGTATGGAGTGATCGCTGCTCTG-3ˊ |
|  | Reverse | 5ˊ-CTGCTCTTGGGTGAAGTTCTCGTAG-3ˊ |
| β-actin | Forward | 5ˊ-GGCCAACCGTGAAAAGATGA-3 |
|  | Reverse | 5ˊ-CAGCCTGGATGGCTACGTACA-3ˊ |

**Supplementary Table 2.** Hematological parameters.

| **Parameter** | **Control** | **Model** | **POD** | **RES** |
| --- | --- | --- | --- | --- |
| **ALT (U/L)** | 45.42±6.41 | 38.24±9.80 | 30.85±20.55 | 49.01±8.28 |
| **AST (U/L)** | 84.65±3.02 | 81.9±7.56 | 77.85±13.36 | 80.3±5.75 |
| **Cr (μmol/L)** | 22.27±11.84 | 35.51±9.12 | 28.22±6.81 | 26.36±15.25 |
| **BUN (mmol/L)** | 7.64±1.25 | 6.65±0.53 | 6.33±0.42 | 6.22±0.76 |


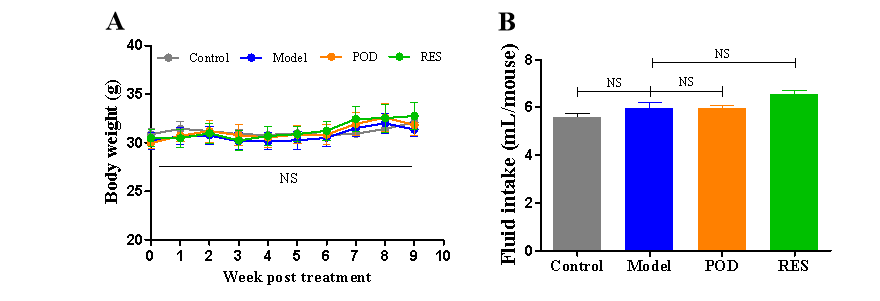


**Supplementary Figure 1.** Effects of POD and RES on body weight and fluid intake in mice fed a high-fructose diet. (A) Body weight. (B) Fluid intake.
